# Topoisomerase 1 Inhibition Promotes Cyclic GMP-AMP Synthase-Dependent Antiviral Responses

**DOI:** 10.1128/mBio.01611-17

**Published:** 2017-10-03

**Authors:** Geneviève Pépin, Charlotte Nejad, Jonathan Ferrand, Belinda J. Thomas, H. James Stunden, Elaine Sanij, Chwan-Hong Foo, Cameron R. Stewart, Jason E. Cain, Philip G. Bardin, Bryan R. G. Williams, Michael P. Gantier

**Affiliations:** a Centre for Innate Immunity and Infectious Diseases, Hudson Institute of Medical Research, Clayton, Victoria, Australia; b Department of Molecular and Translational Science, Monash University, Clayton, Victoria, Australia; c Monash Lung and Sleep, Monash Medical Centre, Clayton, Victoria, Australia; d Institute of Innate Immunity, Biomedical Center, University Hospitals Bonn, Bonn, Germany; e Research Division, Peter MacCallum Cancer Centre, Melbourne, Victoria, Australia; f Department of Pathology, University of Melbourne, Parkville, Victoria, Australia; g CSIRO Health and Biosecurity, Australian Animal Health Laboratory, Geelong, Victoria, Australia; h Centre for Cancer Research, Hudson Institute of Medical Research, Clayton, Victoria, Australia; Brown University

**Keywords:** DNA damage, STING, cGAS, camptothecin, simian virus 40, topoisomerase 1

## Abstract

Inflammatory responses, while essential for pathogen clearance, can also be deleterious to the host. Chemical inhibition of topoisomerase 1 (Top1) by low-dose camptothecin (CPT) can suppress transcriptional induction of antiviral and inflammatory genes and protect animals from excessive and damaging inflammatory responses. We describe the unexpected finding that minor DNA damage from topoisomerase 1 inhibition with low-dose CPT can trigger a strong antiviral immune response through cyclic GMP-AMP synthase (cGAS) detection of cytoplasmic DNA. This argues against CPT having only anti-inflammatory activity. Furthermore, expression of the simian virus 40 (SV40) large T antigen was paramount to the proinflammatory antiviral activity of CPT, as it potentiated cytoplasmic DNA leakage and subsequent cGAS recruitment in human and mouse cell lines. This work suggests that the capacity of Top1 inhibitors to blunt inflammatory responses can be counteracted by viral oncogenes and that this should be taken into account for their therapeutic development.

## IMPORTANCE

Recent studies suggest that low-dose DNA-damaging compounds traditionally used in cancer therapy can have opposite effects on antiviral responses, either suppressing (with the example of CPT) or potentiating (with the example of doxorubicin) them. Our work demonstrates that the minor DNA damage promoted by low-dose CPT can also trigger strong antiviral responses, dependent on the presence of viral oncogenes. Taken together, these results call for caution in the therapeutic use of low-dose chemotherapy agents to modulate antiviral responses in humans.

## OBSERVATION

The ability of innate immune sensors to distinguish self from nonself nucleic acids is essential to host protection. Accordingly, physical segregation between the sensors and the nucleic acids in different cellular compartments is paramount to safeguard aberrant recruitment of immune sensors to self-DNA and self-RNA. As such, cytoplasmic localization of the DNA sensor cyclic GMP-AMP synthase (cGAS) helps minimize sensing of nuclear double-stranded DNA (dsDNA). However, the existence of specific machinery limiting cytoplasmic DNA accumulation indicates that nuclear DNA can find its way to the cytoplasm under steady-state conditions. This is best exemplified with the cases of DNase II and DNase III (TREX1) exonuclease deficiencies, which result in cytoplasmic DNA accumulation and basal engagement of the cGAS-dependent immune response (1). Stimulator of interferon genes (STING) operates downstream of cGAS to activate the transcription factor interferon (IFN) regulatory factor 3 (IRF3) and selectively promote antiviral responses through type I IFN production; it is activated upon binding by cyclic GMP-AMP (2′-5′) (cGAMP), the product of cGAS (2).

Recent evidence suggests that nuclear DNA can be leaked into the cytoplasm upon DNA damage, thereby promoting activation of cGAS-STING signaling (3–8). While such cytoplasmic DNA leakage can be partially attributed to the high doses of genotoxic agents used in some of these studies (3, 4), the reported capacity of nontoxic doses of DNA binding agents such as acriflavine and doxorubicin (Dox) to recruit such cytoplasmic effects and antiviral activities was somewhat unexpected (6, 7). Indeed, short-term treatment with low doses of the genotoxic agents camptothecin (CPT) and topotecan (TPT) reduced induction of several antiviral proteins through translational inhibition (9), arguing against cGAS-STING engagement with these agents. Importantly, CPT and TPT are selective inhibitors of topoisomerase 1 (Top1), while Dox inhibits topoisomerase 2 (Top2) and acriflavine inhibits both (10). Positing that inhibitions of Top1 and Top2 activities have different effects on DNA leakage and cytoplasmic recruitment of cGAS-STING, we investigated the capacity of low-dose CPT to instigate a cGAS-STING antiviral response. We discovered that low-dose CPT can result in strong cGAS/STING-dependent antiviral effects in cells expressing viral oncogenes such as the simian virus 40 (SV40) large T antigen (SV40T).

**FIG 1:**
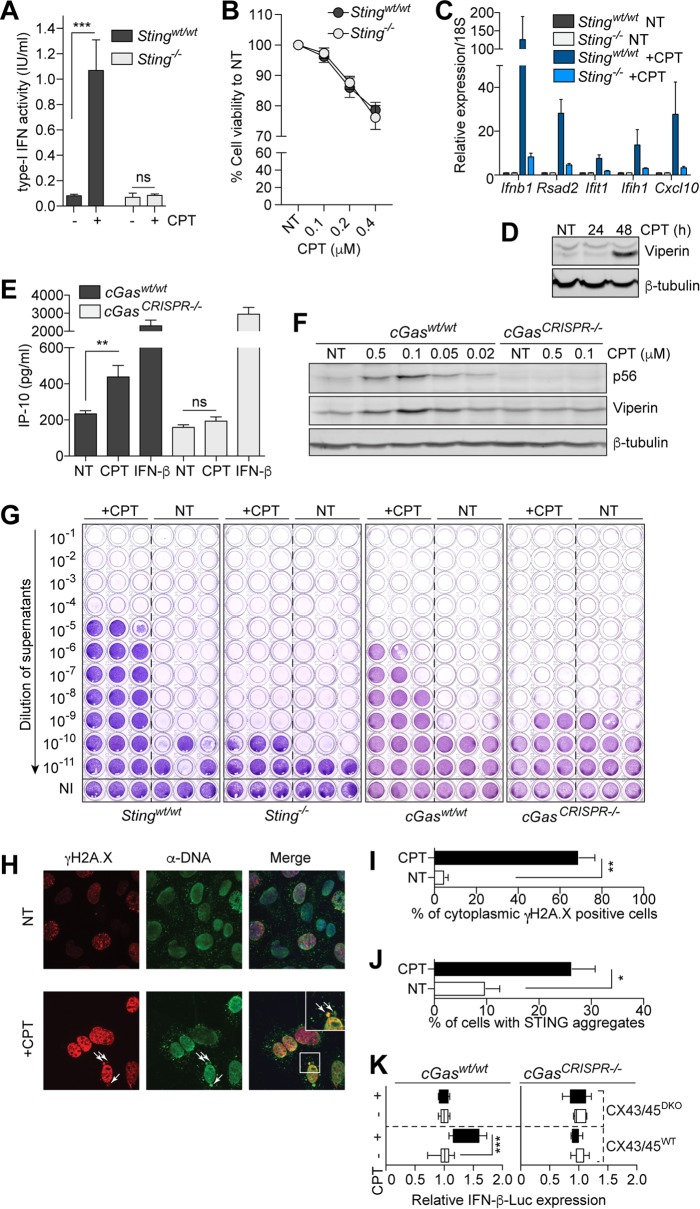
cGAS-dependent activation of antiviral responses by camptothecin (CPT). (A) Type I IFN production by SV40T MEFs from matched wild-type and *Sting*-deficient mice stimulated with 0.1 μM CPT for 48 h, measured by bioassay on LL171 cells (data shown as averages from three independent experiments in biological triplicate ± standard errors of the means). (B) SV40T MEFs from panel A were treated with indicated doses of CPT for 48 h before cell viability analysis with alamarBlue. NT, nontreated. Fluorescence was reported relative to the nontreated condition (data are averages from three independent experiments in biological triplicate ± standard errors of the means). (C) Wild-type and *Sting*-deficient SV40T MEFs were stimulated with 0.1 μM CPT for 48 h, before lysis for RNA purification and reverse transcription-quantitative real-time PCR analysis of ISGs. Gene expression relative to 18S rRNA was further reported relative to the nontreated condition and is averaged from three independent experiments in biological duplicate (±standard errors of the means). (D) Wild-type SV40T MEFs were treated with 0.1 μM CPT for 24 or 48 h and lysed for Western blot analysis of viperin. Data shown are representative of three independent experiments. (E and F) Matched wild-type and *cGas*-deficient SV40T MEFs were stimulated with 0.1 μM CPT (48 h) or 1,000 IU/ml of IFN-β (18 h) (E) or with indicated doses of CPT (48 h) (F), and supernatants and lysates were collected. (E) IP-10 levels were measured by specific enzyme-linked immunosorbent assay. Data shown are averaged from two independent experiments in biological triplicate (±standard errors of the means and significance calculated by the unpaired Mann-Whitney U test are shown). (F) Western blot analysis of p56 and viperin. Data shown are representative of two independent experiments. (G) Matched wild-type and *Sting*-deficient SV40T MEFs or wild-type and *cGas*-deficient SV40T MEFs were treated for 48 h with 0.1 μM CPT prior to 24-h infection in biological triplicate with SFV (multiplicity of infection of 2). Viral titers were assayed with log_10_-fold dilutions on confluent Vero cells as shown. NI, not infected (uninfected cells stained with crystal violet); NT, nontreated. Data shown are representative of a minimum of two independent experiments for each genotype. (H) Immunofluorescence of γ-H2A.X staining (red) and anti-dsDNA (green) and 4′,6-diamidino-2-phenylindole (blue, in merge panels) of wild-type SV40T MEFs incubated with 0.1 μM CPT for 48 h. NT, not treated. White arrows point to cytoplasmic DNA/phospho-γ-H2A.X-positive foci. (I) Percentages of cytoplasmic DNA/phospho-γ-H2A.X-positive cells as shown in panel H; data are averaged from two independent experiments in biological duplicate (±standard errors of the means and significance calculated by the unpaired Mann-Whitney U test are shown). (J) STING-citrine-expressing cells were stimulated with 0.1 μM CPT for 24 h. Percentages of cells exhibiting STING aggregates were quantified; data are averaged from two independent experiments in biological duplicate (±standard errors of the means and significance calculated by the unpaired Mann-Whitney U test are shown). (K) HEK-Sting CX43/45^WT^ and Sting CX43/45^DKO^ cells expressing an IFN-β–luciferase reporter were cocultured with MEFs (matched wild type or *cGas* deficient) pretreated or not with 0.1 μM CPT for 24 h. IFN-β–luciferase expression was reported relative to the nontreated condition for each cell line (data presented are averaged from three independent experiments in biological triplicate, and ±standard errors of the means and significance calculated by the unpaired Mann-Whitney U test are shown). *, *P* ≤ 0.05; **, *P* ≤ 0.01; ***, *P* ≤ 0.001; ns, not significant.

We have recently reported that DNA damage instigated by overexpression of Cre recombinase could engage Sting signaling in SV40T-immortalized mouse embryonic fibroblasts (MEFs) (5). In light of the previous association between Cre genotoxicity and Top1 inhibition (11), we investigated the effect of low-dose CPT (0.1 μM) on the expression of antiviral genes in these cells. Strikingly, 48-h treatment with low-dose CPT induced the production of type I interferon (IFN) by SV40T MEFs while having only a modest impact on cell proliferation (Fig. 1A and B). Critically, type I IFN production upon CPT stimulation was directly reliant on Sting, as it was absent in *Sting*-deficient SV40T MEFs (Fig. 1A). Consequently, IFN-stimulated gene (ISG) expression was also reduced in *Sting*-deficient MEFs (Fig. 1C). Similarly to what we observed with acriflavine treatment of SV40T MEFs (6), ISG induction by low-dose CPT was relatively late, and absent at 24 h, as exemplified by induction of the antiviral protein viperin (encoded by *Rsad2*) after 24 h (Fig. 1D). In line with the activation of Sting by CPT evidenced above, induction of several antiviral proteins (viperin, p56, and IP-10) by CPT was ablated in *cGas*-deficient MEFs (Fig. 1E and F) (6, 12). Accordingly, CPT priming of SV40T MEFs resulted in a strong cGas- and Sting-dependent antiviral effect with a >1,000-fold reduction of viral titers upon Semliki Forest virus (SFV) infection (Fig. 1G).

To provide evidence to support direct engagement of cGas, we first studied the cytoplasmic levels of DNA leaked upon CPT treatment. In agreement with other types of DNA damage in these cells (5, 6), CPT-driven DNA damage significantly increased the proportion of cells displaying colocalized cytoplasmic γ-H2A.X/DNA foci (Fig. 1H and I). STING aggregation was also increased upon CPT stimulation, indicative of cGAMP production (Fig. 1J and data not shown). To directly implicate cGAMP production, we relied on a coculture of the MEFs pretreated with CPT, incubated with human embryonic kidney (HEK) cells expressing the murine Sting and an IFN-β–luciferase reporter (13). Since cGAMP can be transferred between adjacent cells through connexins forming gap-junctions, its production by MEFs can be indirectly measured in recipient human reporter cells which express Sting (6, 13). We found a cGas-dependent induction of the IFN-β–luciferase reporter in HEK cells cocultured with CPT-treated SV40T MEFs (Fig. 1K). This activity of CPT required expression of connexins 43 and 45 in Sting-competent recipient HEK cells (Fig. 1K), thus recapitulating cGAMP activity (13). Altogether, these findings firmly establish the capacity of low-dose CPT to promote cGas-Sting-dependent ISG expression through leakage of DNA into the cytoplasm in SV40T MEFs.

**FIG 2:**
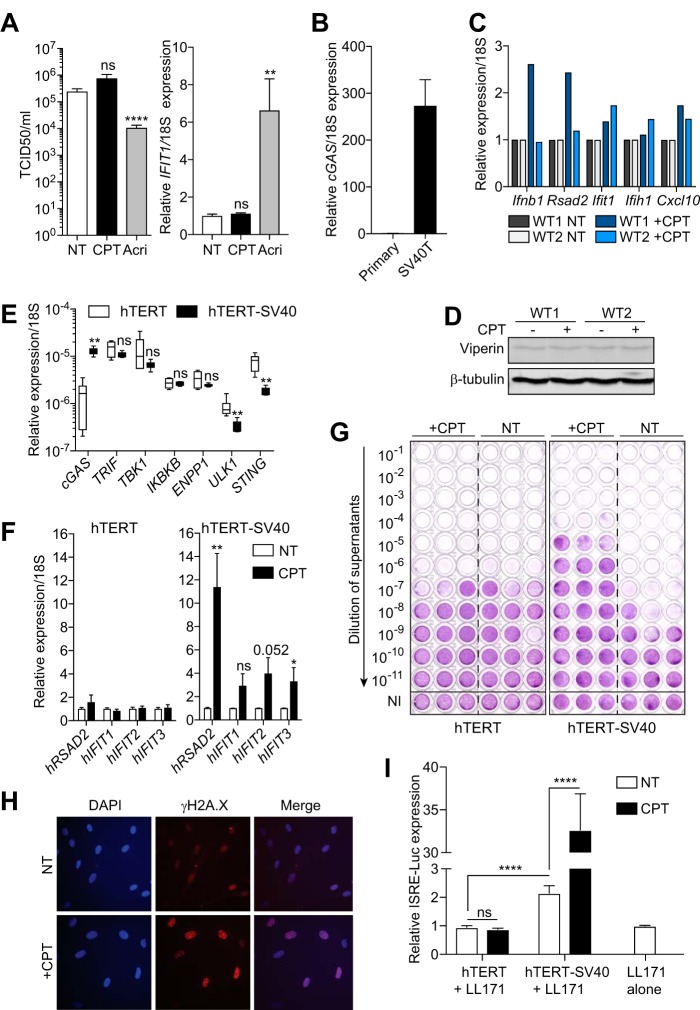
SV40T-dependent activation of antiviral responses by camptothecin. (A) (Left panel) Primary human bronchial epithelial cells (PBECs) were treated with 0.1 μM CPT or 1 μM acriflavine for 72 h prior to infection with rhinovirus 16 (multiplicity of infection of 1) for an additional 24 h, and viral titers were determined by titration on HeLa cells. Titers are averaged from two independent experiments in biological triplicate (±standard errors of the means and significance calculated by the unpaired Mann-Whitney U test relative to nontreated [NT] condition are shown). TCID_50_, 50% tissue culture infective dose. (Right panel) Reverse transcription-quantitative real-time PCR analyses of *IFIT1* from treated PBECs for 72 h prior to infection. Data shown are averaged from three independent experiments in biological duplicate, relative to nontreated cells (±standard errors of the means and significance calculated by the unpaired Mann-Whitney U tests relative to nontreated condition are shown). (B) cGas mRNA expression relative to 18S rRNA measured in two primary wild-type MEF lines compared to WT SV40T MEFs (in biological duplicate). (C and D) Primary wild-type MEFs from two different embryos (WT1 or WT2) were treated with 0.1 μM CPT for 48 h before lysis for reverse transcription-quantitative real-time PCR analysis (C) or viperin Western blot analysis (D). (E) Reverse transcription-quantitative real-time PCR analyses of genes implicated in cGAS-STING sensing in human hTERT fibroblasts and hTERT expressing SV40T. Gene expression relative to 18S rRNA was averaged from three independent experiments in biological duplicate (±standard errors of the means and results of unpaired Mann-Whitney U tests comparing each gene in hTERT SV40T samples to hTERT samples are shown). (F) Reverse transcription-quantitative real-time PCR analyses of selected ISGs in human hTERT fibroblasts (left) and hTERT cells expressing SV40T (right) treated with 0.05 μM CPT for 48 h. Data shown are averaged from three independent experiments in biological duplicate, relative to nontreated cells (±standard errors of the means and significance calculated by the unpaired Mann-Whitney U tests relative to nontreated condition are shown). (G) hTERT fibroblasts with or without SV40T pretreated for 48 h with 0.05 μM CPT were infected for 24 h with SFV (multiplicity of infection of 2), and viral titers were measured on confluent Vero cells as described in the Fig. 1 legend. Data shown are representative of three independent experiments. (H) Immunofluorescence of γ-H2A.X staining (red) and 4′,6-diamidino-2-phenylindole (blue) in hTERT fibroblasts cells incubated for 72 h without infection. NT, not treated. (I) Murine LL171 cells (L929 cells expressing an ISRE-luciferase reporter) were cultured for 18 h in the absence (“LL171 alone”) or presence of hTERT cells or hTERT SV40, pretreated with 0.05 μM CPT for 24 h. ISRE-luciferase expression is shown relative to the LL171-alone condition (data presented are averaged from three independent experiments in biological triplicate, and ±standard errors of the means and significance calculated by unpaired Mann-Whitney U tests are shown). *, *P* ≤ 0.05; **, *P* ≤ 0.01; ****, *P* ≤ 0.0001; ns, not significant.

The capacity of low-dose CPT (0.1 μM) to directly engage a strong antiviral response was unexpected, given the prior report that it inhibited IFN-β-induced genes at similarly low doses (0.5 μM) *in vitro* and displayed potent anti-inflammatory activities in *in vivo* mouse infections with several pathogens [*Staphylococcus aureus* and influenza A (H1N1) virus] (9). To define the biological relevance of our findings in human cells, we tested priming of human primary bronchial epithelial cells (PBECs) with low-dose CPT and compared this to low-dose acriflavine, which we found induced antiviral effects in these cells in previous studies (6). Unexpectedly, while Top1/2 inhibition with acriflavine significantly induced an antiviral effect against rhinovirus (also seen by ISG induction [Fig. 2A, right panel]), Top1 inhibition with CPT failed to do so (Fig. 2A). This lack of responsiveness of PBECs to low-dose CPT, while in agreement with the work from Rialdi et al. (9), led us to hypothesize that our MEF model favored cGas-Sting engagement upon CPT stimulation. Previous work suggests that SV40T expression initiates a low-level DNA damage response promoting type I IFN and ISG expression (14). We speculated that such a low-level IFN response in SV40T MEFs could prime cGas sensing of cytoplasmic DNA through its basal upregulation. In agreement with this, basal *cGas* expression was higher in SV40T MEFs than in primary MEFs (Fig. 2B). Unlike SV40T MEFs, CPT treatment of primary MEFs failed to robustly induce viperin protein levels and only marginally (less than 3-fold) induced ISGs analyzed in different primary MEF lines (including *Ifnb1*) (Fig. 2C and D), supporting a role for SV40T in the activity of CPT on cGas in MEFs. To further implicate SV40T expression in the response to CPT, we next studied the responsiveness of cGAS-competent human telomerase reverse transcriptase (hTERT)-immortalized human foreskin fibroblasts (referred to here as human fibroblasts [6]) and their variant additionally expressing the large SV40 T antigen (15) to low-dose CPT. Importantly, analysis of expression of a panel of genes involved in cGAS-STING signaling revealed that while cGAS expression was increased ~10-fold, expression of STING and ULK1 (16) was decreased ~4- and ~2-fold, respectively, upon SV40T expression in these human fibroblasts, but none of the other components of the pathway were changed (e.g., TBK1, IKBKB, TRIF, and ENPP1) (Fig. 2E). Although SV40T decreased STING expression, the negative activity of ULK1 on STING signaling (16) led us to posit that the net effect of SV40T expression would rather potentiate cGAS sensing, similarly to what we saw in SV40T MEFs. In line with this, while human fibroblasts did not show any ISG response to CPT exposure, their SV40T-expressing counterparts displayed a significant induction of ISGs (Fig. 2F). Accordingly, CPT pretreatment significantly protected human fibroblasts against SFV infection depending on their SV40T expression (Fig. 2G). Importantly, human fibroblasts without SV40T exhibited increased levels of nuclear γ-H2A.X staining upon CPT stimulation, confirming that low-dose DNA damage occurred in these cells (Fig. 2H). Finally, coculture of CPT-pretreated human fibroblasts with L929 mouse fibroblasts stably expressing an IFN-stimulated responsive element (ISRE)–luciferase reporter (referred to as LL171 cells) confirmed transfer of cGAMP-like activity from SV40T human fibroblasts but not their parental line (Fig. 2I) (13).

### Conclusion.

Our work establishes that Top1 inhibition by low-dose CPT can, at least in cells expressing SV40T, induce a strong cGAS-dependent antiviral immune response. Although SV40 and its large T antigen are not really a concern in humans (17), our finding suggests that other, similar viral oncogenes may also potentiate leaking of damaged DNA to the cytoplasm, even when low-dose genotoxic agents are used. In support of this, cytoplasmic DNA was also reported in HeLa cells after low-dose Dox treatment (7), possibly relating to the expression of human papillomavirus 18 (HPV18) E6/E7. In light of the prevalence of certain latent viral oncogenes in humans (Epstein-Barr virus carries the EBNA-1 oncogene and is, for instance, present in 90% of adults [18]), our work highlights the need for caution when translating the use of Top1 inhibitors as inflammatory suppressors in the context of acutely exacerbated immune responses (such as in bacterial and viral infections), as has been recently proposed (9).

Interestingly, and in accord with the concept that low-dose CPT does not induce antiviral responses in normal cells (9), low-dose CPT exposure failed to promote ISG expression in human fibroblasts, even though low-level DNA damage was observed. Conversely, low-dose acriflavine (1 μM) treatment of human PBECs and fibroblasts did promote antiviral responses in these cells (6). These observations support the concept that immune sensing of the moderate DNA damage generated by Top1 inhibition markedly differs from that of Top2 inhibition. Indeed, CPT inhibits the religation of single-stranded DNA (ssDNA) after Top1 cleavage, which predominantly involves the recruitment of ATR/Chk1 (with a negligible role for ATM [19]). Conversely, Top2 inhibition results in double-stranded DNA breaks promoting the rapid autophosphorylation of ATM (7). Recent studies have suggested that Top2 inhibition with low-dose Dox (1.5 to 3 μg/ml) could induce a type I IFN response independently of cGAS, through ATM activation (7). Critically, cytoplasmic DNA leakage was not required for the ATM response (7). These observations clearly call for more studies to help delineate how Top1 and Top2 inhibition controls antiviral responses, although high doses of DNA damage are likely to result in cytoplasmic DNA leaking to the cytoplasm, independent of the type of damage incurred and the presence of viral oncogenes (3, 4).

How SV40T expression increases cGAS sensing of damaged DNA is not clear; however, SV40T is known to raise basal ISG expression through ATR activation (14). Consequently, SV40T expression directly increases the sensitivity of cGAS to cytoplasmic DNA by basally augmenting cGAS levels in MEFs (cGAS being an ISG [20]) and human fibroblasts (Fig. 2). However, SV40T expression may also favor cytoplasmic DNA accumulation. Indeed, we failed to observe any cytoplasmic DNA in primary MEFs or human fibroblasts treated with low-dose CPT (data not shown). Given the delayed cGAS recruitment (>24 h) after DNA damage, our observations with SV40T-expressing cells may be related to their cell cycle progression through mitosis, a phenomenon recently shown to promote cytoplasmic DNA leaking and cGAS activation in cancer cells (8). We note that Ahn et al. previously reported the activation of a cGas-dependent IFN response to low-dose (0.5 μM) CPT in primary MEFs (3) but that these observations were based on *Trex1*-deficient MEFs, which exacerbate cytoplasmic DNA accumulation. Irrespective of its mode of action, our data demonstrate that SV40T expression potentiates cGAS sensing of damaged DNA, similarly to what is observed in *Trex1*-deficient cells (3). Interestingly, and in contrast to its role on cGAS, SV40T expression decreased STING mRNA levels in human fibroblasts while also reducing levels of ULK1. Given that ULK1 phosphorylates STING and suppresses IRF3 function when STING is activated by cyclic dinucleotides (16), SV40T appears to exert both positive and negative effects on STING function, the net effect of which may depend on the modalities of STING activation. Indeed, in addition to cGAMP, STING can be directly activated by viral envelopes and liposomes (21, 22) commonly used in DNA transfection. It is therefore a possibility that STING engagement independent of cGAMP may be dampened by SV40T expression. This last point may help to explain the discrepancies between our findings on SV40T potentiation of cGAS sensing of endogenous DNA and those of Lau et al., which rather described an inhibitory action of SV40T on STING detection of liposome-transfected DNA (23).

With the rapid development of drug resistance by viruses such as influenza virus, broad-spectrum antiviral molecules are a timely therapeutic option. Given the established capacity for the cGAS-STING pathway to inhibit a wide range of viruses, molecules engaging this pathway are likely candidates for such broad antiviral activities (24). Our current work, along with that of other groups, suggests that low-level DNA damage has the capacity to instigate potent antiviral activities, which could be harnessed to protect at-risk populations from pandemic infections (6, 7). Nonetheless, this study demonstrates that the mechanism of how DNA damage engages antiviral responses is more complex than previously proposed. While further studies are warranted to better understand how genotoxic agents recruit the immune system, cGAS-STING-specific agonists may prove to be more adequate broad-spectrum antivirals *in vivo*.

### Cell culture.

All mouse embryonic fibroblast (MEF) lines used in this study were immortalized with the SV40 large T antigen using pSG5-SV40-LT-Ag (gift from D. Huang, Walter and Eliza Hall Institute of Medical Research, Melbourne, Australia) (6, 12). *Sting*-deficient SV40T MEFs and their matched wild type were previously reported (6). *cGas*^*CRISPR−/−*^ MEFs (referred to as *cGas*-deficient MEFs) were generated by clustered regularly interspaced short palindromic repeat(s) (CRISPR) and were derived from a parental *cGas*^*wt/wt*^-immortalized MEF line (used as a matched control) (12). Human fibroblasts (hTERT-BJ1 cells) stably expressing SV40 (large and small T antigens), together with an oncogenic allele of HRAS (HRASG12V), referred to as hTERT-SV40 here, were previously reported (15). HEK Sting and HEK Sting CX43/45^DKO^ cells stably expressing the murine Sting fused to an N-terminal mCherry tag, together with LL171 reporter cells (L929 expressing an interferon-stimulated response element [ISRE]–luciferase), were all previously described (13). HEKs, MEFs, and LL171 and Vero cells were grown in Dulbecco’s modified Eagle’s medium (DMEM; Thermo Fisher) supplemented with 10% sterile fetal bovine serum (Thermo Fisher) and 1× antibiotic-antimycotic (Thermo Fisher) (referred to as complete DMEM). Human fibroblasts (hTERT-BJ1 cells, referred to as hTERT cells) were grown in complete DMEM supplemented with sodium pyruvate (Thermo Fisher). When needed, cells were treated with acriflavine (Sigma catalog no. A8126), camptothecin (Sigma catalog no. C9911; resuspended in dimethyl sulfoxide [DMSO]), or recombinant mouse IFN-β at 1,000 IU/ml (gift from N. A. de Weerd, A. Matthews, and P. J. Hertzog, Hudson Institute). alamarBlue cell viability assays were performed according to the manufacturer’s protocol (Thermo Fisher).

Additional methods are described in Text S1 in the supplemental material.

10.1128/mBio.01611-17.1 TEXT S1  Supplemental methods. Download TEXT S1, PDF file, 0.1 MB. Copyright © 2017 Pépin et al. 2017 Pépin et al. This content is distributed under the terms of the Creative Commons Attribution 4.0 International license.
