# Supplementary material for: Topoisomerase 1 Inhibition Promotes Cyclic GMP-AMP Synthase-Dependent Antiviral Responses
Source: mBio. 2017 Oct 3;8(5):e01611-17. doi: 10.1128/mBio.01611-17 (PMC5626974; doi:10.1128/mBio.01611-17)
Supplement: TEXT S1 [file mbo005173520s1.pdf]

Supplementary methods

Cytokine analyses: For murine type I IFN assay, LL171 reporter cells (L929 expressing an interferon stimulated response element (ISRE)-Luciferase) ***(1)*** were seeded the day before at 20,000 cells per well of a 96 well-plate in 100 µL of complete media. 100 µL of neat supernatant from MEF treated with 0.1 µM camptothecin for 48 h was added to the LL171. Serial dilution of recombinant murine IFNβ was used to prepare a standard curve ***(2)***. Luciferase activity was analysed 6 h later as previously reported ***(3)***. Murine IP-10 production in supernatant of MEFs treated with 0,1 µM camptothecin for 48 h or 1000 IU of recombinant type I IFN for 24 h was quantified using Mouse CXCL10/IP-10/CRG-2 Duo Set ELISA (Dy466) from R&D systems according to the manufacturer's protocol.

Western blotting. MEFs were seeded in 24 well plates at a density of ~25,000 cells per well for 48 h and 50,000 cells per well for 24 h. Cell lysates were analysed as previously described ***(3)***. Protein detection was carried out using 1:1000 mouse monoclonal anti-Viperin (MaP.VIP | MABF106, Millipore), rabbit anti-mouse p56 (gift from G. Sen, Cleveland Clinic, Cleveland, Ohio, USA) or mouse monoclonal anti-beta-tubulin (TU-06 | ab7792, Abcam). Finally, conjugated secondary antibodies with Alexa Fluor® 680 dye (Life Technologies) or IRdye800 (Rockland) were used to image the proteins at 700 or 800 nm with an Odyssey scanner (LI-COR).

Infections: *Semliki Forest Virus (SFV) infection*: 120,000 MEFs or 80,000 hTERT/ hTERT-SV40 cells were seeded in 24-well plates 48 h after camptothecin treatment (0.1 µM for MEF and 0.05 µM for hTERT), and left to adhere for several hours, prior to infection with SFV in complete DMEM (multiplicity of infection (MOI) of 2 – as determined by plaque forming units in Vero cells) (each condition was carried out in biological triplicate), as previously described ***(3)***. *Rhinovirus infection*: Primary human bronchial epithelial cells (PBEC) were obtained and cultured as

described previously ***(4)***. The Monash Health and Monash Medical Centre Human Research Ethics Committee approved the studies; consent was obtained from all subjects, and studies were conducted in accordance with the approved guidelines. Briefly, PBECs were obtained from bronchial brushings during routine bronchoscopy and cultured under submerged conditions on collagen-coated flasks (MP Biomedicals) in supplemented bronchial epithelial growth medium (BEGM; Lonza). When PBEC reached 80% confluency they were treated with Acriflavine 1 µM or camptothecin 0.1 µM for 3 days. Cells were then washed and infected with rhinovirus 16 in BEGM without hydrocortisone at a MOI of 1 for 1 hr, then washed and incubated in BEGM without hydrocortisone for 24 hr. Supernatants and cell lysates were collected for analysis. Viral titres in supernatants were determined by titration on Ohio HeLa cells, as described previously ***(5)***.

Reverse transcription quantitative real-time PCR (RT-qPCR). Total RNA was purified from cells using the ISOLATE II RNA Mini Kit (Bioline). For mRNA quantification, cDNA was synthesized from isolated RNA using the High-Capacity cDNA Archive kit (Life Technologies) according to the manufacturer's instructions. RT-qPCR was carried out with the SensiFAST™ SYBR® Hi-ROX Kit (Bioline) or the power SYBR mix (Life Technologies) on the HT7900 RT-PCR system (Life Technologies). Each PCR was carried out in technical duplicate. Mouse and human 18S were used as reference genes. Each amplicon was sequence-verified and used to generate a standard curve for the quantification of gene expression (used in each run). Melting curves were analysed in each run to confirm specificity of amplification. Components of the cGAS-STING pathway were selected based on previous reports (e.g. TBK1, IKBKB ***(6)***, TRIF ***(7)***, ENPP1 ***(8)***, ULK1 ***(9)***). The primers used were the following: Mouse 18S: mRn18s-FWD GTAACCCGTTGAACCCCATT; mRn18s-REV CCATCCAATCGGTAGTAGCG; Mouse Ifit1: mIfit1-RT-FWD GAGAGTCAAGGCAGGTTTCT; mIfit1-

RT-REV TCTCACTTCCAAATCAGGTATGT; Mouse Rsad2: mRsad2-FWD CTGTGCGCTGGAAGGTTT; mRsad2-REV ATTCAGGCACCAAACAGGAC; Mouse Ifnb1: mIfnb1-FWD CCCTATGGAGATGACGGAGA; mIfnb1-REV CCCAGTGCTGGAGAAATTGT; Mouse Cxcl10: mCxcl10-FWD GCTGCCGTCATTTTCTGC; mCxcl10-REV CACTGGGTAAAGGGGAGTGA; Mouse Ifih1: mIfih1-FWD TCTTGGACACTTGCTTCGAG; mIfiH1-REV TCCTTCTGCACAATCCTTCTC; Mouse cGas: mcGas-FWD TGAACATGTGAAGATTTCTGCTCC; mcGas-REV TGACTCAGCGGATTTCCTCG; Human RSAD2: hRSAD2-RT-FWD TGGTGAGGTTCTGCAAAGTAG; hRSAD2-RT-REV GTCACAGGAGATAGCGAGAATG; Human IFIT1: hIFIT1-FWD TCACCAGATAGGGCTTTGCT; hIFIT1-REV CACCTCAAATGTGGGCTTTT; Human IFIT2: hIFIT2-RT-FWD TTATTGGTGGCAGAAGAGGAAG; hIFIT2-RT-REV CCTCCATCAAGTTCCAGGTG; Human IFIT3: hIFIT3-FWD CATAAAAGCACAGACCTAACAGC; hIFIT3-REV CAGGGAATTCTTGGTGACCTC; Human TBK1: hTBK1-FWD AAGCCGGAAGTGTCCTGAGT; hTBK1-REV ACCAGTTTTCTTATGTCTTCCACGA, Human IKBKB: hIKBKB-FWD CTTCCCTGACAACGCAGACA; hIKBKB-REV AATCTGCTCACCTGTTTCCTGA; Human ENNPP1: hENPP1-FWD AGATGAAAGACCACACTTTTACACT; hENPP1-REV AGGTTCAGGCATCTGTGCAA; Human TRIF: hTRIF-FWD GGATCCCTGATCTGCTTGGG; hTRIF-REV GAATGTCGAAGGCGCTAGGA; Human ULK1: hULK1-FWD GTTCCAAACACCTCGGTCCT; hULK1-REV CCAACTTGAGGAGATGGCGT; Human STING: hSTING-FWD GGCATCAAGGATCGGGTTTA; hSTING-REV TGTCCGGCAGAAGAGTTTG; Human cGAS: hcGAS-FWD CACGTATGTACCCAGAACCC; hcGAS-REV GTCCTGAGGCACTGAAGAAAG; Human 18S: h18S-FWD CGGCTACCACATCCAAGGAA; h18S-REV GCTGGAATTACCGCGGCT;

Immunofluorescence. Cells plated on coverslips were fixed in 10% formalin following camptothecin treatment for 48 h (0.1 µM for MEF and 0.05 µM for hTERT). For STING aggregation count (Figure 1J), MEF were transiently transfected with pEF-BOS-hSTING-citrine plasmid for 48h prior to be plated on coverslips and treated with 0.1 µM of CPT for 24 h.

Detection of γ-H2A.X and cytoplasmic DNA was carried out as previously described ***(3)***. Briefly, following cell permeabilisation and blocking, DNA staining was performed using 1/50 dilution of anti-DNA (#AC-30-10 Novus Biological) for 1 h. Following PBS washes, A 1/250 dilution of Alexa Fluor® 647 conjugated rabbit monoclonal anti-PhosphoHistone-H2A.X (Ser139) (9720, Cell Signaling) was incubated for 1 h, together with 1/1000 dilution of goat anti-mouse AlexaFluor 568 IgM antibody (used as a secondary antibody for anti-DNA detection). Coverslips were mounted on slides using *Prolong Diamond Antifade Mountant with DAPI* (Life Technologies, P36971). Confocal imaging was performed using API DeltaVision Widefield (Figure 1I) or Olympus FluoView FV1200 Laser Scanning Confocal Microscope (Figure 1J). Percentages of cytoplasmic positive phospho-γ-H2A.X cells (Figure 1I) or STING aggregates (Figure 1J) were determined by counting cells with cytoplasmic bright foci (Figure 1I) or aggregates (Figure 1J) (using 20X-40X objective), reported to the total number of cells (determined with DAPI staining). Data are from two independent experiments with two coverslips per condition per experiment. Overall, at least 150 cells have been counted per condition per independent experiment. Image analyses were performed using ImageJ v1.49.

Co-culture studies. HEK-Sting were transfected with Interferon-β-Luc reporter plasmid (pLuc-IFN-β, a kind gift from K. Fitzgerald, University of Massachusetts). Briefly 0.4 µg of DNA was reverse-transfected in 500,000 HEK-Sting (CX43/45^{WT} or CX43/45^{DKO}) cells using Lipofectamine 2000. After 4 h incubation, the cells were co-cultured in a new plate with MEF (WT or *cGas*$^{CRISPR-/-}$ MEFs) previously treated with 0.1 µM camptothecin for 24 h (with a ratio of around 85,000 HEKs for 50,000 MEFs) in a 24 well-plate. Luciferase activity was analysed 18 h later as previously reported ***(3)***. For co-culture studies with LL171 reporter cells, hTERT and hTERT-SV40 were previously treated with 0.05 µM camptothecin for 24 h prior to their transfer in a new 24 well-plate and co-

culture with LL171 cells for 18 h (ratio of 70,000 hTERT for 70,000 LL171).

Statistical analyses. Statistical analyses were carried out using Prism 6 (GraphPad Software Inc.). Two-tailed unpaired non-parametric Mann-Whitney U tests were used to compare pairs of conditions. Symbols used: ns: not significant, * P≤0.05, ** P≤0.01, *** P≤0.001, **** P≤0.0001.

References

1. Uze G, Di Marco S, Mouchel-Vielh E, Monneron D, Bandu MT, Horisberger MA, Dorques A, Lutfalla G, Mogensen KE (1994) Domains of interaction between alpha interferon and its receptor components. J Mol Biol 243 (2):245-257. doi:10.1006/jmbi.1994.1651
2. Stifter SA, Gould JA, Mangan NE, Reid HH, Rossjohn J, Hertzog PJ, de Weerd NA (2014) Purification and biological characterization of soluble, recombinant mouse IFNbeta expressed in insect cells. Protein Expr Purif 94:7-14. doi:10.1016/j.pep.2013.10.019
3. Pepin G, Ferrand J, Honing K, Jayasekara WS, Cain JE, Behlke MA, Gough DJ, Williams BRG, Hornung V, Gantier MP (2016) Cre-dependent DNA recombination activates a STING-dependent innate immune response. Nucleic Acids Res 44 (11):5356-5364. doi:10.1093/nar/gkw405
4. Thomas BJ, Porritt RA, Hertzog PJ, Bardin PG, Tate MD (2014) Glucocorticosteroids enhance replication of respiratory viruses: effect of adjuvant interferon. Sci Rep 4:7176. doi:10.1038/srep07176
5. Thomas BJ, Lindsay M, Dagher H, Freezer NJ, Li D, Ghildyal R, Bardin PG (2009) Transforming growth factor-beta enhances rhinovirus infection by diminishing early innate responses. Am J Respir Cell Mol Biol 41 (3):339-347. doi:10.1165/rcmb.2008-0316OC
6. Abe T, Barber GN (2014) Cytosolic-DNA-mediated, STING-dependent proinflammatory gene induction necessitates canonical NF-kappaB activation through TBK1. J Virol 88 (10):5328-5341. doi:10.1128/JVI.00037-14
7. Wang X, Majumdar T, Kessler P, Ozhegov E, Zhang Y, Chattopadhyay S, Barik S, Sen GC (2016) STING Requires the Adaptor TRIF to Trigger Innate Immune Responses to Microbial Infection. Cell Host Microbe 20 (3):329-341. doi:10.1016/j.chom.2016.08.002
8. Li L, Yin Q, Kuss P, Maliga Z, Millan JL, Wu H, Mitchison TJ (2014) Hydrolysis of 2'3'-cGAMP by ENPP1 and design of nonhydrolyzable analogs. Nat Chem Biol 10 (12):1043-1048. doi:10.1038/nchembio.1661
9. Konno H, Konno K, Barber GN (2013) Cyclic dinucleotides trigger ULK1 (ATG1) phosphorylation of STING to prevent sustained innate immune signaling. Cell 155 (3):688-698. doi:10.1016/j.cell.2013.09.049
